# The impact of different multi-walled carbon nanotubes on the X-band microwave absorption of their epoxy nanocomposites

**DOI:** 10.1186/s13065-015-0087-2

**Published:** 2015-03-04

**Authors:** Bien Dong Che, Bao Quoc Nguyen, Le-Thu T Nguyen, Ha Tran Nguyen, Viet Quoc Nguyen, Thang Van Le, Nieu Huu Nguyen

**Affiliations:** National Key Laboratory of Polymer and Composite Materials, Ho Chi Minh City University of Technology (HCMUT), Vietnam National University, 268 Ly Thuong Kiet, District 10, Ho Chi Minh City, Vietnam; Faculty of Materials Technology, Ho Chi Minh City University of Technology, Vietnam National University, 268 Ly Thuong Kiet, District 10, Ho Chi Minh City, Vietnam; Materials Technology Key Laboratory (Mtlab), Ho Chi Minh City University of Technology, Vietnam National University, 268 Ly Thuong Kiet, District 10, Ho Chi Minh City, Vietnam

**Keywords:** Radar absorbing materials (RAMs), Carbon nanotubes, Nanocomposites, X-band microwave absorption, Epoxy composites

## Abstract

**Background:**

Carbon nanotube (CNT) characteristics, besides the processing conditions, can change significantly the microwave absorption behavior of CNT/polymer composites. In this study, we investigated the influence of three commercial multi-walled CNT materials with various diameters and length-to-diameter aspect ratios on the X-band microwave absorption of epoxy nanocomposites with CNT contents from 0.125 to 2 wt%, prepared by two dispersion methods, i.e. in solution with surfactant-aiding and via ball-milling.

**Results:**

The laser diffraction particle size and TEM analysis showed that both methods produced good dispersions at the microscopic level of CNTs. Both a high aspect ratio resulting in nanotube alignment trend and good infiltration of the matrix in the individual nanotubes, which was indicated by high Brookfield viscosities at low CNT contents of CNT/epoxy dispersions, are important factors to achieve composites with high microwave absorption characteristics. The multi-walled carbon nanotube (MWCNT) with the largest aspect ratio resulted in composites with the best X-band microwave absorption performance, which is considerably better than that of reported pristine CNT/polymer composites with similar or lower thicknesses and CNT loadings below 4 wt%.

**Conclusions:**

A high aspect ratio of CNTs resulting in microscopic alignment trend of nanotubes as well as a good level of micro-scale CNT dispersion resulting from good CNT-matrix interactions are crucial to obtain effective microwave absorption performance. This study demonstrated that effective radar absorbing MWCNT/epoxy nanocomposites having small matching thicknesses of 2–3 mm and very low filler contents of 0.25-0.5 wt%, with microwave energy absorption in the X-band region above 90% and maximum absorption peak values above 97%, could be obtained via simple processing methods, which is promising for mass production in industrial applications.

**Graphical Abstract:**
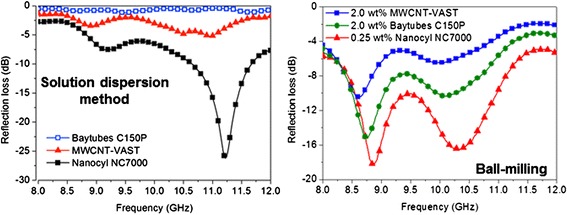
Comparison of the X-band microwave reflection loss of epoxy composites of various commercial multi-walled carbon nanotube materials.

## Background

Carbon nanotubes (CNTs) as nano-fillers in polymer matrix composites have captivated much interest from many industries and research groups, owing to the impressive physical properties of CNTs such as high elastic modulus as well as high thermal and electrical conductivities. CNT-filled composites have proven great potential for commercial applications for aerospace, transportation, automotive and electronic industries. CNTs as fillers offering a good conductive network in polymer matrices can also result in enhanced dielectric loss, which causes attenuation of microwave energy. Thus, there have been abundant studies on CNT-filled polymer nanocomposites as microwave absorbers and electromagnetic shielding materials gaining remarkable attention in both civil and military applications [1-7].

Due to strong van der Waals forces, CNTs tend to agglomerate. The ability to effectively minimize the amount of CNT entangled bundles and disperse the nanotubes in polymer matrices influences nearly all relevant properties of the composites. The effects of CNT dispersibility via different dispersion methods, such as melt mixing using extruders, solvent processing by means of centrifugation, ultrasonication, surfactant treatment and chemical modification of CNTs, on the mechanical, thermal and electrical properties of CNT composites have been well-addressed [8-21]. While an excellent dispersion is essential for effectively reinforcing polymer matrices [22], a good conductivity requires both a good distribution of dis-entangled CNT agglomerates and conglomeration of CNTs in an anisotropic morphology necessary for constitution of a conductive network [23]. The shape anisotropy and spatial orientation of nano-fillers in nanocomposites could have a crucial influence on the electrical conductivity [24]. It has been reported that strong CNT-polymer interactions or increased compatibility of CNTs to the polymer matrix, which enhance polymer-wrapping around CNTs, could decrease the electrical conductivity [15,23]. It has also been found that multi-walled carbon nanotube (MWCNT)/polymer composite films with CNT agglomerations at the micro-scale have higher electrical conductivity than those with uniformly dispersed CNTs [25]. Depending on the synthesis and processing conditions, the properties of MWCNTs from different producers can vary enormously. Several works have compared the mechanical and thermal properties and electrical conductivity of polymer composites of various commercial CNTs. For example, Pötschke and coworkers [16,26] compared the nanotube dispersity via light microscopy, the mechanical and electrical characteristics, associated with the extrusion feeding conditions, of twin-screw extruded polypropylene composites of two types of MWCNTs, namely Baytubes C150P and Nanocyl NC7000 having different mean length-to-diameter ratios, bulk densities and agglomerate strength. Three-roll mill processed epoxy composites of Baytubes C150P and Nanocyl NC7000 with equal filler contents showed different electrical resistivities [27]. Castillo et al. [28] compared five MWCNT materials from different suppliers with various aspect ratios on the electrical, mechanical and glass transition behavior of polycarbonate-based nanocomposites. Rahaman et al. [29] reported the different electrical properties of polyethylene nanocomposites of three types of commercial MWCNTs with different aspect ratios. Ball-milling treatment of the as-synthesized Nanocyl NC700 MWCNTs to alter the CNT length and bulk density resulting in a change in the electrical conductivity of their melt-mixed polypropylene-based nanocomposites has been observed by Menzer et al. [30]. Gojny et al. [23] investigated the different thermal and electrical conductivities of epoxy composites of different single-walled, double-walled and multi-walled CNTs as well as amino-functionalized CNTs from various producers. The effects of MWCNTs with different properties on mechanical reinforcement as well as on the electrical percolation threshold of composites based on other types of polymers, such as high density polyethylene and polyamide, have also been shown in other works [22,31,32].

However, a good conductivity does not necessarily correspond to an effective microwave absorbing performance, which needs to satisfy not only dielectric loss requirements, but also importantly the impedance matching condition [33,34].

The formation of a dense interconnected CNT network can give rise to enhanced dielectric loss but should not make the material substantially reflective [35]. The microwave absorption properties of CNT-filled nanocomposites depend on not only the intrinsic electrical conductivity of CNTs, the interactions among CNTs, matrix-CNT interactions but also CNT clustering, which results in polarization phenomena and hence frequency dependence of effective permittivity [33]. In this aspect, CNT properties like nanotube type, length, diameter, bulk density, surface quality, purity, the size and strength of agglomerates, which are dependent on the CNT synthesis conditions, affect significantly the dispersity of CNTs throughout the polymer, the tendency of CNT re-clustering, and thereby the microwave absorption performance.

Numerous studies researched the dependence of polymer composite performance on the grade of MWCNT filler as mentioned above, while fewer investigations on the influence of CNTs on the microwave absorbing efficiency of CNT-polymer composites were reported [36-39].

On the other hand, for practical applications, 0.5-0.6 wt% CNT loadings are normally the optimal CNT contents for not compromising the composite fracture strength [16,40], and a thin composite thickness of a few milimeters is often preferred for radar absorbing composite coatings on metal or textile substrates. Thin composites also give the advantages of lightweight and cost-effectiveness. It has been shown in the literature that pristine CNT/polymer nanocomposites satisfying both a low CNT content below 0.6 wt% and a small composite thickness below 4 mm have not achieved a reflection loss below −10 dB desirable for radar absorbing applications. Thus, either high CNT loadings of 4–30 wt%, large composite thicknesses or the synthesis of CNT-metallic magnetic particle hybrids have been employed in order to enhance the microwave absorption efficiency of CNT/polymer composites [33,35,41-54]. However, CNT characteristics, a crucial factor besides the processing conditions that can change significantly the microwave absorption behavior, have not been addressed.

Therefore, in this article, the microwave absorbing properties in the X-band (8–12 GHz) region of epoxy-based nanocomposites of three different commercial MWCNT materials from diverse producers, i.e. Baytubes C150P (Bayer Material-Science AG, Germany), Nanocyl NC7000 (Nanocyl S.A., Belgium) and MWCNT-VAST (VAST, Vietnam) are compared. The two methods of processing in solution with surfactant-aiding and via ball-milling were employed, and composites having different MWCNT contents were fabricated. An investigation of the dispersibility of the different MWCNTs in solution and in the epoxy matrix via transmission electron microscopy (TEM), particle sizing and Brookfield viscosity measurements was performed, and was correlated to the electrical conductivity and microwave absorption behavior of their composites.

## Results and discussion

### Characterization of dry MWCNT powders

**Figure 1:**
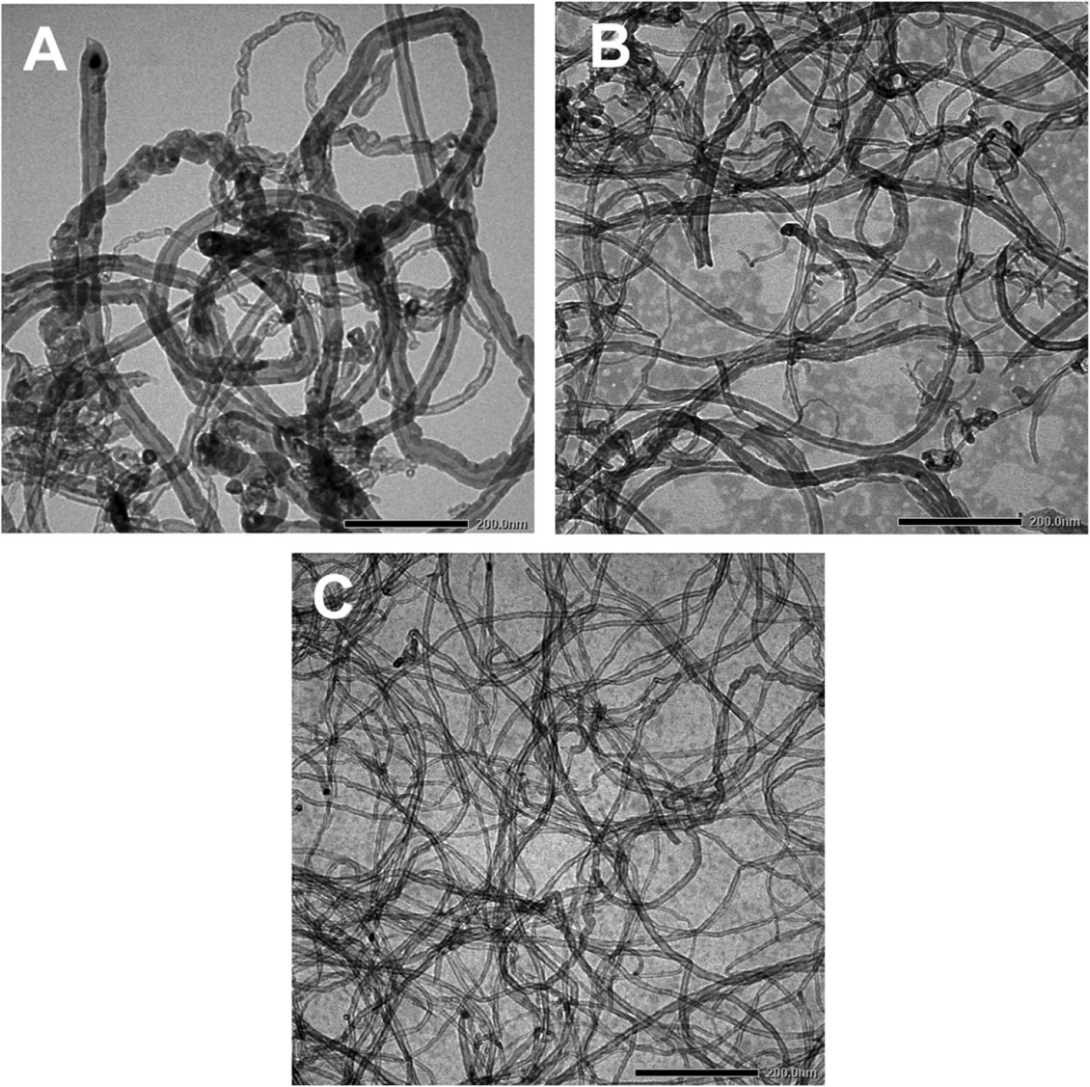
**TEM micrographs of the MWCNT powders (scale bar: 200 nm): (A) MWCNT-VAST, (B) Baytubes C150P, and (C) Nanocyl NC7000.**

TEM images of the different pristine MWCNT powders are shown in Figure 1. The TEM micrographs highlight the increasing CNT average diameters of Nanocyl NC7000, Baytubes C150P and MWCNT-VAST, in this order. Nanocyl NC7000 CNTs have significantly thinner wall as well as more uniform diameter distribution, as compared to Baytubes C150P and MWCNT-VAST.

**Figure 2:**
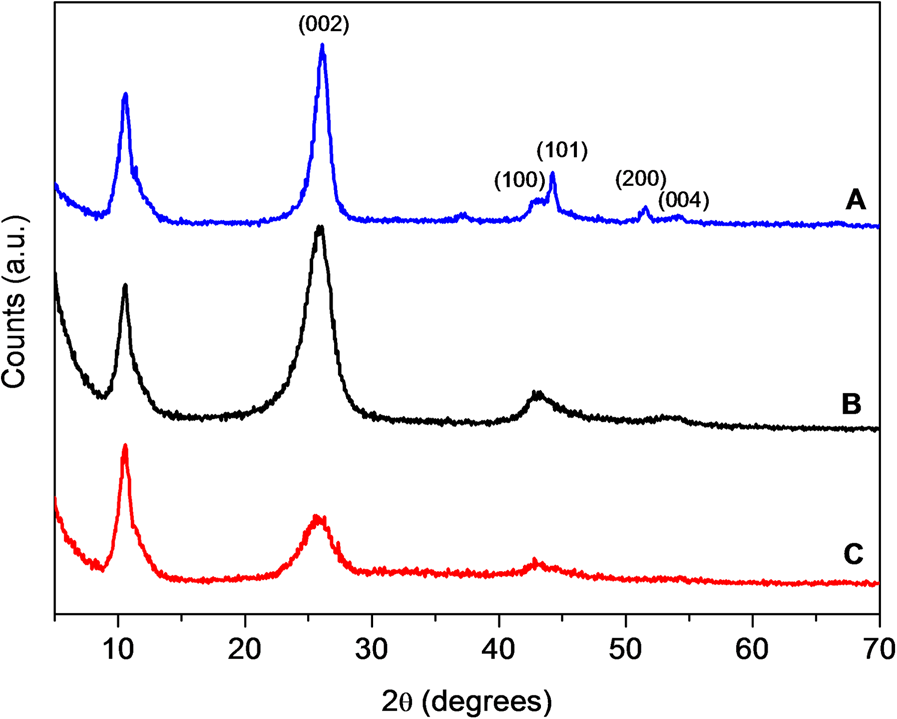
**XRD patterns of the MWCNT powders: (A) MWCNT-VAST, (B) Baytubes C150P, and (C) Nanocyl NC7000.**

Figure 2 compares the XRD patterns of the used MWCNT materials, which show almost the same diffraction (002) peak at 2θ of 26.7 − 26° corresponding to a d-spacing between graphene sheets of 3.42 − 3.46 Å, as well as the (100) peak at 43 Å related to the in-plane graphitic structure. The decreases of inter-wall distance d_(002)_ ranging from 3.42, 3.43 to 3.46 Å and FWHM of the (002) peak ranging from 2.3, 2.2 to 1.2° for Nanocyl NC7000, Baytubes C150P and MWCNT-VAST (Table 1), respectively, are indicative of increasing levels of graphitic structures [55]. Compared to Nanocyl NC7000 and Baytubes C150P, MWCNT-VAST exhibited a (101) peak at 44.1°, which originates from a lateral correlation between graphite layers [56]. In addition, all the samples show a peak at 2θ = 10.5° corresponding to a d spacing of 8.4 Å, which is similar to the characteristic diffraction peak of graphite oxide [57,58]. Another difference in the XRD patterns of the MWCNTs is the intensity of the (002) diffraction peak. Because the contribution of the intratube structure to the (002) peak increased with wall number [59], the much lower intensity of the (002) peak of Nanocyl NC7000 could be related to the considerably thinner wall compared to those of Baytubes C150P and MWCNT-VAST, which was confirmed by TEM.

**Table 1:** **The XRD interlayer spacing d and width of the (002) peak, and the Raman band characteristics of the MWCNT powders**

**Sample**	**XRD**		**Raman**		
	**d**_**(002)**_ **(Å)**	**FWHM**_**(002)**_ **(** ^**o**^ **)**	**I**_**D**_ **/I**_**G**_	**FWHM**_**G**_ **(cm** ^**−1**^ **)**	**FWHM**_**D**_ **(cm** ^**−1**^ **)**
Nanocyl NC7000	3.46	2.3	1.84	75.4	66.5
Baytubes C150P	3.43	2.2	1.95	75.4	65.6
MWCNT-VAST	3.42	1.2	1.5	65.3	53.4

**Figure 3:**
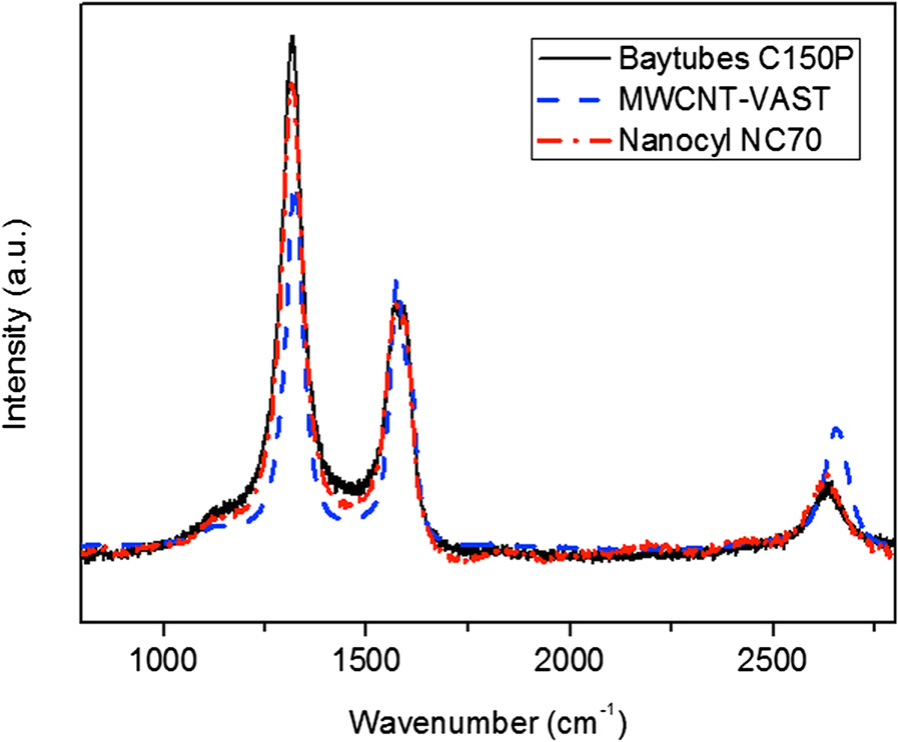
**Raman spectra of the MWCNT powders: MWCNT-VAST, Baytubes C150P, and Nanocyl NC7000.**

The structural ordering of the MWCNTs was additionally analyzed by Raman spectroscopy, which gives information on the defects (D band at around 1320 cm^−1^), in-plane vibration of sp^2^ carbon atoms (G band at around 1580 cm^−1^) and the stacking orders (G’ band at around 2643 cm^−1^) [60]. The intensity of the G band (I_G_) does not depend on the lattice defect density, whereas the D band intensity (I_D_) increases and the G’ band intensity (I_G’_) decreases as defect density increases. As shown in Figure 3 and Table 1, the smaller intensity ratio of D to G band (I_D_/I_G_) and full width at half maximum (FWHM) of the G band, as well as the slightly higher I_G_’/I_G_ of MWCNT-VAST compared to the other two MWCNT materials indicate a higher degree of graphitization, which is in agreement with the XRD result. We also found that the FWHM_D_ of the D-band of MWCNT-VAST was smaller than those of Nanocyl NC7000 and Baytubes C150P. Such prominent difference in the Raman characteristic bands arises from the significantly larger CNT diameter and thicker wall of MWCNT-VAST. These observations are similar to previous reports which showed that the D band intensity and FWHM_D_ were larger for MWCNTs with smaller diameters and smaller number of graphene layers, as a result of large strain in the tube walls leading to breakdown of lattice translational symmetry [61].

### MWCNT/epoxy nanocomposites prepared via the solution dispersion method

#### Particle size distribution of MWCNTs in ethanol dispersions

In the solution dispersion method, composites of MWCNTs and epoxy resin were fabricated by mixing the epoxy resin with nanotubes pre-dispersed in ethanol, followed by solvent evaporation afterward. The dispersion of MWCNTs in ethanol was conducted under ultrasonication, with the addition of 0.05 wt% of sodium dodecyl benzene sulfonate (NaDDBS), which is one of ionic surfactants commonly used to reduce the aggregative tendency of CNTs in water [62].

The initial swelling of CNT agglomerates by solvent infiltration and interaction has to be considered as a crucial precondition to obtain a good dispersion of CNTs inside the polymer matrix, which is a critical aspect for achieving good absorbing materials. Thus, investigations of the dispersability of different MWCNT materials in ethanol, via assessment of their average aggregated size and size distribution, were performed by laser diffraction particle size analysis. It has been reported that Nanocyl NC7000 and Baytubes C150P particles in ultrasonicated aqueous surfactant dispersions had rod-like shapes, as indicated by dynamic light scattering [63]. It should be noted that the mean particle diameter obtained by this method does not refer directly to nanotube size, but to their agglomerate size, which is an average between tube bundle length and diameter.

**Figure 4:**
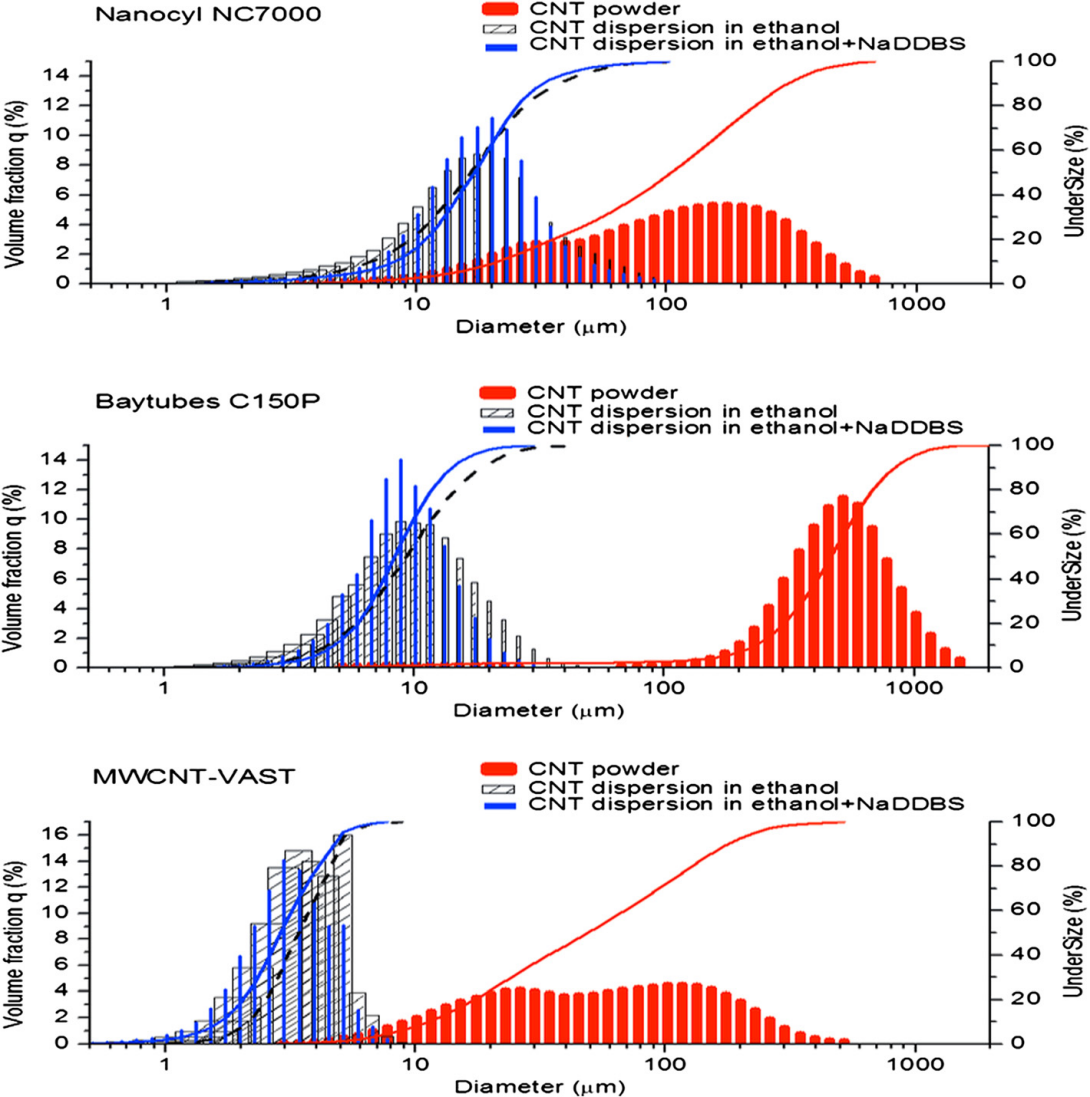
**Size distributions of the MWCNT powders, and their ultrasonicated dispersions in ethanol without and with 0.05 wt% of NaDDBS.** Ethanol was used as the dispersant.

**Figure 5:**
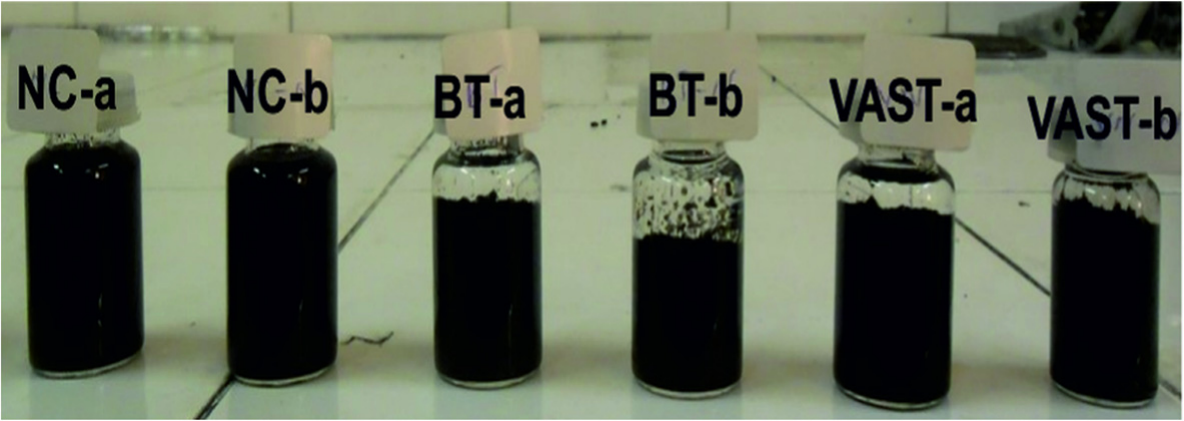
**States of the sonicated MWCNT dispersions in ethanol, with (−a) and without NaDDBS (−b) after 36 hours: Nanocyl NC7000 (NC-a and -b), Baytubes C150P (BT-a and -b), and MWCNT-VAST (VAST-a and -b).**

As shown in Figure 4 and Table 2, all the MWCNTs powders existed in aggregated forms with bimodal and large size distributions. Sonication of MWCNTs in ethanol at 55°C for 60 min was sufficient to significantly reduce the agglomerate size, resulting in 3.5 − 20 μm monomodal distributions. The use of the NaDDBS surfactant only slightly lowered the agglomerate size and size distribution, suggesting that the best dispersed state of the MWCNTs was obtained. The particle size analysis revealed the largest agglomerates in the powder form of Baytubes C150P, whereas in the sonicated dispersion state the agglomerate size of the MWCNTs was correlated to their length-to-diameter aspect ratio. While the Baytubes C150P and MWCNT-VAST nanotubes were dispersed in the medium as individuals, with the average size close to the tube lengths, the Nanocyl NC7000 nanotubes seemed to cluster with an average bundle size of around 20 μm attributed to their larger length-to-diameter aspect ratio. This is in accordance with previously reported data that the Nanocyl NC7000 nanotubes were much longer than Baytubes C150P as revealed by TEM analysis [26,28,64,65]. Moreover, the ethanol dispersions of Nanocyl NC7000, both with and without NaDDBS, appeared to be the most stable, remaining homogeneous after 36 hours, whereas the dispersions of both Baytubes C150P and MWCNT-VAST partially sedimented (Figure 5). The dispersions of Baytubes C150P were least stable. The sedimentation of both Baytubes C150P and MWCNT-VAST dispersions was slightly reduced with the assistance of the NaDDBS surfactant.

**Table 2:** **Mean diameters (μm) of the MWCNTs obtained by laser diffraction particle size analysis with ethanol as dispersant**

	**Powder**	**Dispersion in ethanol**	**Dispersion in ethanol with 0.05 wt% of NaDDBS**
Nanocyl NC7000	137.4	19.6	19.2
Baytubes C150P	501.3	10.6	9.0
MWCNT-VAST	75.8	3.5	3.0

#### Microwave absorption of MWCNT/epoxy nanocomposites via the solution dispersion method

To study the microwave absorption performance of the MWCNT/epoxy composites, the reflection loss of the prepared metal-backed single-layered composites was measured in the X-band.

**Figure 6:**
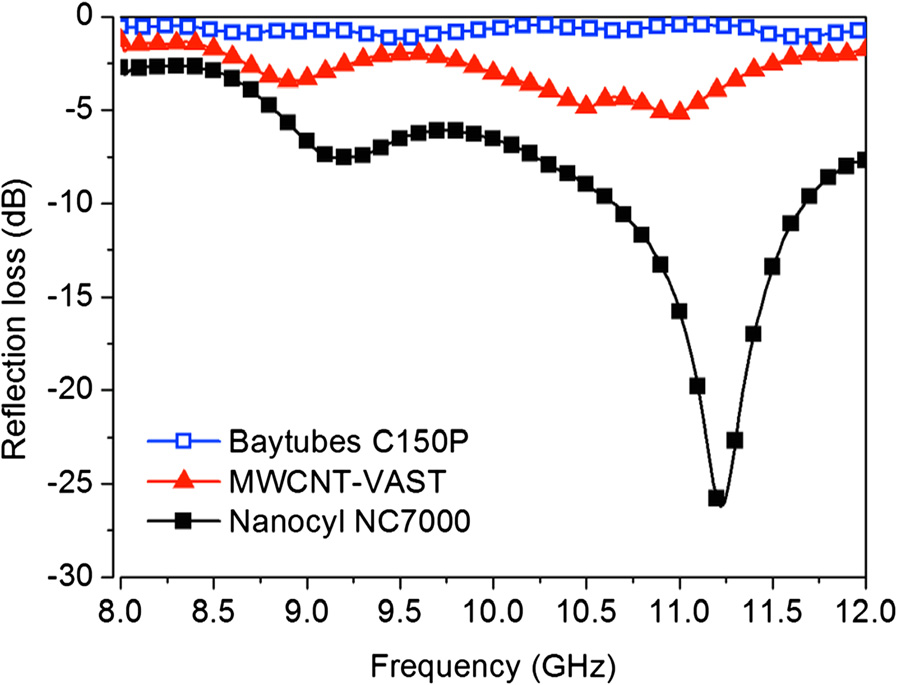
**Reflection loss versus frequency of 2 mm thick MWCNT/epoxy composites prepared via the solution dispersion method, with 0.5 wt% of CNT content and 0.05 wt% of NaDDBS.**

The frequency dependences of the microwave absorbing characteristics in the X-band region of 2 mm thick MWCNT/epoxy composites with 0.5 wt% of CNT content prepared using the ethanol surfactant dispersions of the different MWCNT materials are compared in Figure 6. With an equal CNT filler content, the composite of Nanocyl NC7000 showed the highest microwave absorption, exhibiting a reflection loss peak with the maximum value of 26.1 dB at 11.2 GHz. The microwave absorption maximum of the composite of MWCNT-VAST reached 5 dB, corresponding to 70% microwave energy absorption, while microwave absorption was insignificant for the composite of Baytubes C150P. The difference in the microwave absorption behavior of the composites was not correlated to the aggregate size of the CNT dispersion, but seems to be in accordance with the CNT dispersion stability. Despite the fact that Nanocyl NC7000 existed as larger agglomerates, at a low CNT loading of 0.5 wt%, only its composite achieve a reflection loss value in the X-band frequency region above 10 dB, which is desirable for an effective RAM.

### MWCNT/epoxy nanocomposites prepared via the ball-milling dispersion method

The influence of the MWCNT materials on the microwave absorption properties of their epoxy composites prepared via ball-milling dispersion of nanotubes in the resin matrix was further investigated. From a practical point of view, this dispersion method is advantageous especially for mass production, since it requires no addition of a solvent and thereby no solvent evaporation as well as ultrasonication and mechanical stirring. For all the MWCNT materials used, CNT loadings in the matrix for radar-absorbing study were limited to maximum 2 wt%, in order to ensure the composite structural integrity and mechanical properties.

#### Brookfield viscosity

The viscosity of MWCNT/epoxy dispersions has a correlation with the spatial and orientation of CNTs in the matrix, which could reflect the quality of the dispersion to a certain extent. The viscosities of different ball-milled MWCNT/epoxy dispersions with the various MWCNT materials and different nanotube contents are summarized in Table 3. Generally, the viscosity increased with increasing CNT loading content. It was observed that at equal CNT loadings, the epoxy resin containing Nanocyl NC7000 had the highest viscosity, followed by that of Baytubes C150P. The considerably higher viscosity of the Nanocyl NC7000/epoxy dispersions suggests a better dispersion of CNTs and stronger interaction between the nanotubes and the polymer matrix compared to Baytubes C150P and MWCNT-VAST [66], which could be attributed to the higher nanotube aspect ratio of Nanocyl NC7000. It was also found that there was a correlation between the upper limited viscosity of the MWCNT/epoxy dispersions, which was about 150000 cP, and the maximum CNT content in order to maintain a uniform distribution of the nanotubes as well as a good microwave absorption ability of the cured composite. For instance, we observed that above 0.75 wt% of Nanocyl NC7000 when the viscosity exceeded 150000 cP, nanotubes started to conglomerate in the epoxy matrix. At the same time, the microwave absorption of the Nanocyl NC7000/epoxy composite with 1 wt% of CNT content was significantly decreased to below the absorption level of 70% of microwave energy, despite the increase in the electrical conductivity as compared to the composites with lower nanotube loadings (data not shown).

**Table 3:** **Brookfield viscosity values measured for the epoxy resin and different ball-milled MWCNT/epoxy dispersions**

**Sample**	**CNT content (wt%)**	**Viscosity (cP)**
Epoxy resin^a^	0	832
Nanocyl NC7000/epoxy^a^	0.25	15200
Nanocyl NC7000/epoxy^a^	0.5	75200
Nanocyl NC7000/epoxy^a^	0.75	149000
Nanocyl NC7000/epoxy^a^	1.0	272000
Baytubes C150P/epoxy^a^	1.0	44000
Baytubes C150P/epoxy^a^	2.0	131000
MWCNT-VAST/epoxy^a^	1.0	2300
MWCNT-VAST/epoxy^a^	2.0	22400

^a^containing 20 wt% of the RD 108 diluent.

#### Microwave absorption properties

Regarding the microwave absorption mechanism, the MWCNTs in the epoxy composites can absorb the microwave energy and attenuate the radiation via the interaction between interior electrons and exterior microwave radiation. On the other hand, the defects in MWCNTs can also act as polarization centers and contribute to strong microwave absorption, attributed mainly to the dielectric relaxation [33,34].

The microwave absorbing properties of the prepared single-layered RAMs were explained with the help of the characteristic electromagnetic parameters by using the Equation (1) and (2) [34], are related in this manner:

1 $$ {Z}_{in}={Z}_0\sqrt{\frac{\mu_r}{\varepsilon_r}} tanh\left[\frac{j2\pi }{c}\sqrt{\mu_r{\varepsilon}_r}fd\right] $$

2 $$ RL=20lo{g}_{10}\left|\frac{Z_{in}-{z}_0}{Z_{in}+{Z}_0}\right| $$

where *Z*_*in*_ is the normalized input impedance at free space and material interface, *Z*_0_ is the characteristic impedance of free space, *μ*_*r*_ and *ε*_*r*_ are respectively the complex relative permeability and permittivity of the material, c is the velocity of light, f is the frequency and d is the sample thickness, *RL* is the reflection loss which is related to the relative impedance mismatch between the shield’s surface and propagating wave.

Besides the dielectric loss requirements, the impedance matching condition (where *Z*_*in*_ is close to *Z*_0_) is important to obtain a good microwave absorption.

As to be shown below, the prepared MWCNT-epoxy composites exhibited CNT content and frequency dependence of the microwave absorbing characteristics, which is attributed mainly to dielectric loss of the composites [50,52].

**Figure 7:**
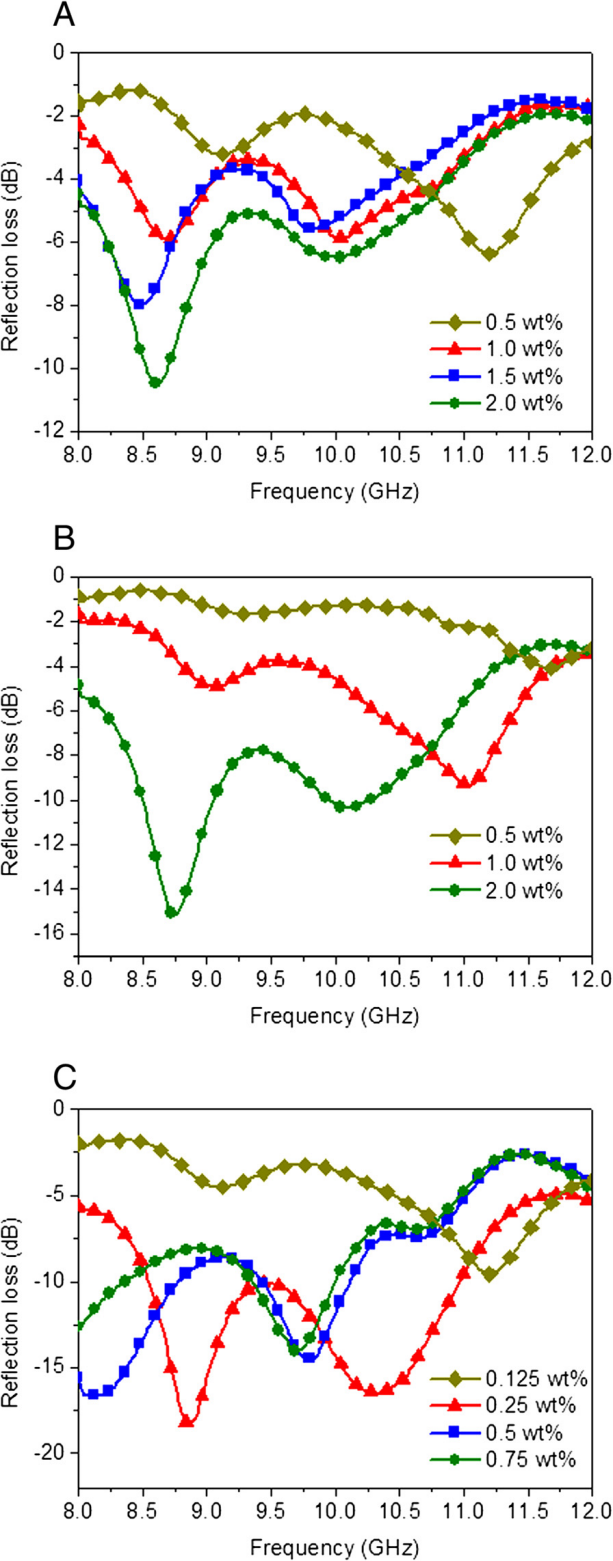
**Reflection loss versus frequency of 3 mm thick MWCNT/epoxy composites with different CNT contents prepared via the ball-milling dispersion method, using various MWCNT materials: (a) MWCNT-VAST, (b) Baytubes C150P, and (c) Nanocyl NC7000.**

As revealed in Figure 7, the epoxy composites of the different MWCNT materials show the same trend in the microwave absorption behavior as a function of CNT content, by which the maximum reflection loss peaks in the X-band region shifted to lower frequencies with increasing CNT content. For the composites of Baytubes C150P and MWCNT-VAST, the microwave absorption increased with CNT content up to 2 wt%, which was the maximum CNT loading to maintain relatively homogeneous distributions with insignificant aggregation of nanotubes. The increase in microwave absorption with CNT content could be attributed to the enhancement of dielectric loss tangent, the factor mainly contributing to the attenuation of microwave energy of carbon nanofiller composites [50,52]. In the case of Nanocyl NC7000, the maximum microwave absorption was obtained at 0.25 wt% CNT. Increasing the CNT content to 0.5 and 0.75 wt% led to slight decreases of maximum reflection loss values, which was due to the increased reflectivity of the composites caused by CNT clustering.

**Figure 8:**
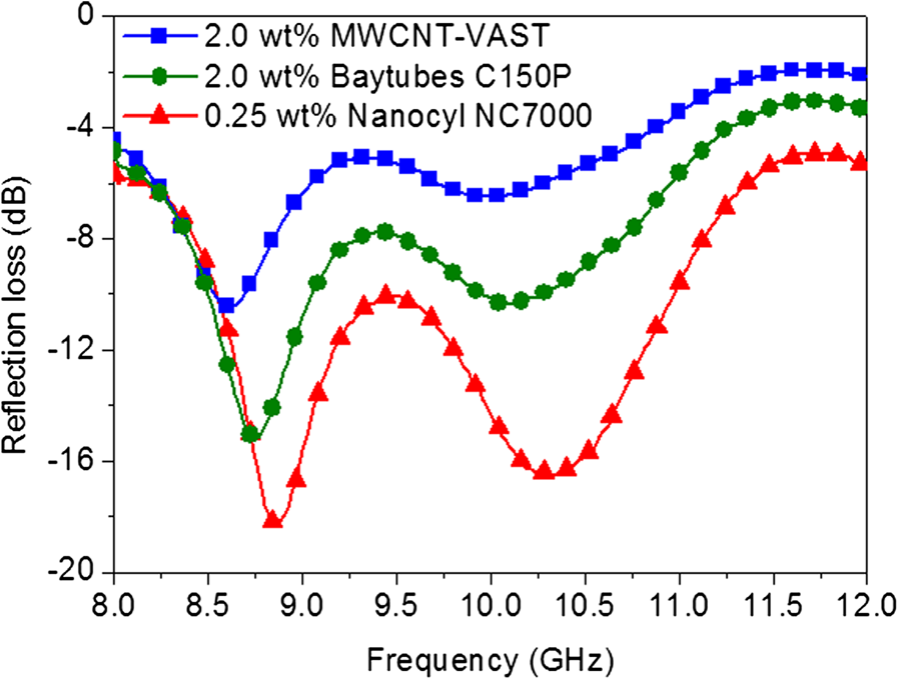
**Comparison of the best microwave absorption performances of 3 mm thick MWCNT/epoxy composites prepared via the ball-milling method using different MWCNT materials: 2 wt% of MWCNT-VAST, 2 wt% of Baytubes C150P, and 0.25 wt% of Nanocyl NC7000.**

In a comparison of the best microwave absorption performances obtained for the composites of the different MWCNT materials (Figure 8), it was observed that the epoxy composites showed reflection loss peaks at similar frequency ranges, i.e. a peak at 8.5-9 and the other at 10–10.5 GHz., but with significantly different reflection loss values. The composite of Nanocyl NC7000 possessed the best microwave absorption at a very low CNT content of only 0.25 wt%, showing maximum reflection loss peaks of 16.5 dB at 10.3 GHz and 18.4 dB at 8.8 GHz. Only at a high CNT content of 2 wt%, the Baytubes C150P could achieve reflection loss above 10 dB, with the maximum peaks of 15.0 dB at 8.7 GHz and 10.5 dB at 10.1 GHz. On the other hand, the 2 wt% MWCNT-VAST composites exhibited the lowest microwave absorption with the maximum peaks of 10.5 dB at 8.6 GHz and 6.5 dB at 10.0 GHz.

It should be emphasized that with a thickness of only 3 mm and low CNT contents, i.e. 2 wt% for Baytubes C150P and 0.25 wt% for Nanocyl NC7000, these composites showed reflection loss values much better than other pristine CNT/polymer composites with similar or lower thicknesses and CNT loadings below 4 wt% reported in the literature. For instance, the MWCNT/epoxy nanocomposite with 20 wt% CNT loading and 1.2 mm thickness reported by Che et al. [41] had a reflection loss of less than 2 dB. Thus, to gain desirable microwave absorption performance of pristine CNT/polymer nanocomposites, high CNT contents were utilized in many other studies. Fan et al. [35] applied twin-screw extrusion and sand-milling to prepare CNT/PET and CNT/varnish composites with 4 and 8 wt% of CNTs and thicknesses of 2 and 1 mm, showing reflection loss peaks at 7.6 and 15.3 GHz with maximum values of 17.61 dB and 24.27 dB, respectively. Liu et al. [50] prepared 2 mm thick CNT/polyurethane nanocomposites with 5 wt% of single-walled CNTs through solution mixing in dimethylformamide followed by slow drying, giving a maximum absorbing value of 22 dB at 8.8 GHz. In other studies on MWCNT/paraffin composites at a substantially high CNT loading of 20 wt%, the maximum absorbing values of the pristine CNT composites reported by Lin et al. [42,44] did not reach the acceptable limit above 10 dB, whereas those by Zhang et al. [45,46] achieved maximum peaks of 22 dB in the X-band region. Helical and worm-like MWCNT/paraffin composites with 30 wt% CNTs and 2.8-3 mm thicknesses have been reported to exhibit maximum reflection loss values of about 26 dB at 7–8 GHz [51]. The nanocomposites of synthesized twin carbon nanocoils in paraffin were prepared obtained maximum reflection loss values above 10 dB in the X-band region at carbon nanocoil contents of 15–22 wt% and matching thicknesses of 3–3.5 mm [52]. Bhattacharya et al. [48] prepared a 2 mm thick unmodified MWCNT/polyurethane nanocomposite at a 30 wt% CNT loading through solution blending using mechanical stirring, with the maximum reflection loss of 16.03 dB at 10.99 GHz. MWCNT/epoxy nanocomposites with CNT loadings, matching thicknesses and maximum reflection loss of 0.5 wt%, 9 mm, 25 dB at 11 GHz as well as 5 wt%, 3 mm, 18 dB at 8 GHz, respectively, have also been reported [53,54].

In addition, it was also found that such difference in the microwave absorption behavior of the composites of Nanocyl NC7000, Baytubes C150P and MWCNT-VAST did not correspond to their different electrical conductivities (Table 4). The 2 wt% MWCNT-VAST composite had a significantly lower electrical conductivity than those of the composites using the other two types of MWCNTs. Normally, the formation of a dense interconnected CNT network can increase the electric properties [33,49]. This facilitates the enhancement of dielectric loss for microwave absorbers [33,49], as long as the high CNT content does not make the material too reflective [35]. Despite the better microwave absorption performance of the 0.25 wt% Nanocyl NC7000 composite, its conductivity was lower as compared to the Baytubes C150P composite.

**Table 4:** **Electrical conductivities of 3 mm thick MWCNT/epoxy composites prepared via the ball-milling method with 2 wt% of MWCNT-VAST, 2 wt% of Baytubes C150P and 0.25 wt% of Nanocyl NC7000**

	**MWCNT content (wt%)**	**Electrical conductivity (10** ^**5**^ **S/cm)**
Nanocyl NC7000	0.25	3.87
Baytubes C150P	2	5.46
MWCNT-VAST	2	<0.005

### TEM analysis

**Figure 9:**
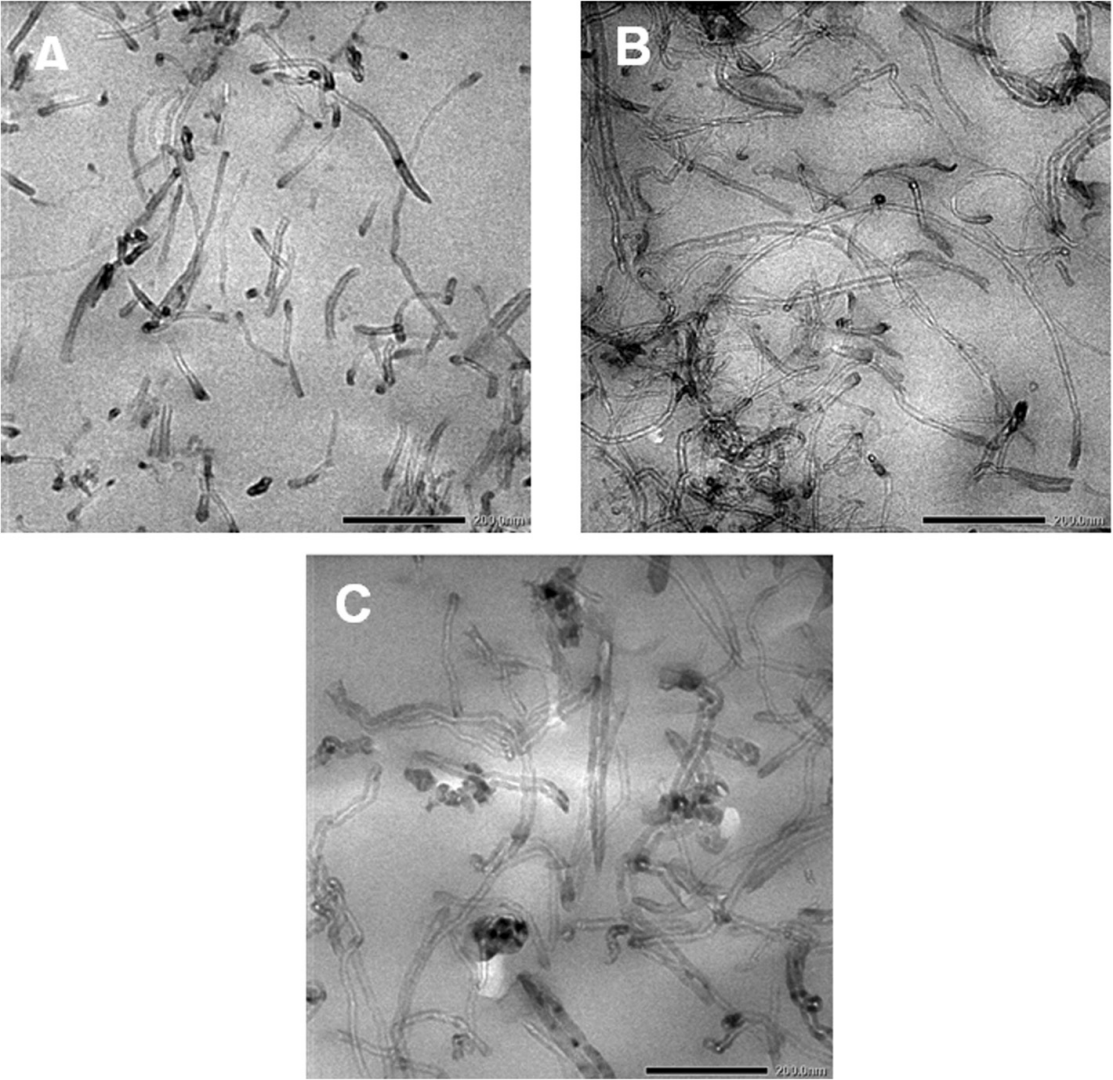
**TEM micrographs of 3 mm thick MWCNT/epoxy nanocomposites prepared using the ball-milling method with (A) 0.25 wt% of Nanocyl NC7000, (B) 2 wt% of Baytubes C150P and (C) 2 wt% of MWCNT-VAST. Scale-bar: 200 nm.**

In addition, the TEM micrographs of the composites of the different MWCNT materials at CNT loadings giving the optimal microwave performance were compared. It is worth noted that the low specific density and the good separation of Nanocyl NC7000 nanotubes could result in a large apparent volume fraction, as compared to the other CNTs for the same mass content. As shown in Figure 9, in the composite of Baytubes C150P there was the existence of a small fraction of CNT aggregates as entangled clusters, which seems to stem from the high packing density of the primary agglomerates of the CNTs, whereas the Nanocyl NC7000 and MWCNT-VAST nanotubes were mostly dis-entangled and dispersed relatively homogeneously in the matrix. Moreover, compared with Baytubes C150P and MWCNT-VAST, the Nanocyl NC7000 nanotubes exhibited a tendency of being aligned in the same directions, which is mainly attributed to the higher length-to-diameter aspect ratio of the Nanocyl NC7000 CNTs. Both the higher aspect ratio and thinner wall of Nanocyl NC7000 resulted in a larger surface area to volume ratio [67] and thus a larger CNT re-agglomeration tendency because of van der Waals and Coulomb attractions [13,68], as well as a larger viscosity shear effect leading to higher MWCNT orientations [69,70]. Hence, a good dispersion of CNTs exhibiting an anisotropic morphology, with a certain aspect ratio, of aligned nanotubes is crucial to achieve an effective microwave absorption. On the other hand, it is possible that the longer MWCNT-VAST CNTs were more damaged during the ball-milling process, giving rise to the worse microwave absorption properties of nanocomposites collated to Baytubes C150P.

## Conclusion

Three different commercially available carbon nanotube materials were studied with regard to the microwave absorption properties of their epoxy composites prepared using the solution mixing and ball-milling dispersion methods. The correlation of the microwave absorption performance of the composites with the CNT dispersability in the matrix and CNT characteristics could indirectly be indicated, to a certain extent, by the CNT agglomerate size in ethanol surfactant solutions, as well as the viscosity of the ball-milled CNT/epoxy dispersions. For all the CNT materials used, the spectra of the reflection loss versus frequency showed the presence of two minima. This phenomenon has been observed for the epoxy composites filled with porous carbon fibers, and was ascribed to the combination of absorption and interference of the microwaves [71].

The difference in microwave absorption of the composites of the different MWCNT materials did not correspond to the trend in the difference of the electrical conductivities. The best microwave absorption behavior was found for the composite of Nanocyl NC7000, even at a much lower CNT content as compared to Baytubes C150P and MWCNT-VAST. It was found that a high aspect ratio of CNTs resulting in microscopic alignment trend of nanotubes as well as a good level of micro-scale CNT dispersion resulting from good CNT-matrix interactions are crucial to obtain effective microwave absorption performance. Especially, Nanocyl NC7000, with a small mean tube diameter, thin tube wall, high length-to-diameter aspect ratio and uniform size distribution, proved to be the most suitable MWCNT material for the fabrication of effective MWCNT/polymer composite RAMs at very low CNT contents and small composite thicknesses. For instance, up to 2 wt% of Baytubes C150P was required to give a relatively effective 3 mm thick RAM with reflection loss above 10 dB. It is noted that the radar absorbing performance of the epoxy composites of Nanocyl NC7000 obtained in this work is considerably better than that of pristine CNT/polymer composites with similar or lower thicknesses and CNT loadings below 5 wt% reported so far [33].

Through this study, we demonstrate for the first time to the best of our knowledge, that by suitable selection of the MWCNT material, effective radar absorbing MWCNT/epoxy nanocomposites having small matching thicknesses of 2–3 mm and very low filler contents of 0.25-0.5 wt%, with microwave energy absorption in the X-band region above 90% and maximum absorption peak values above 97%, could be obtained via simple processing methods, which is promising for mass production in industrial applications.

## Experimental

### Materials

Baytubes C150P (Bayer Material-Science AG, Germany), Nanocyl NC7000 (Nanocyl S.A., Belgium) and MWCNT-VAST (VAST, Vietnam) multiwalled carbon nanotube (MWCNT) materials, all synthesized via the chemical vapor deposition (CVD) method, were used as received. The properties of the MWCNT materials as given in the corresponding data sheets are shown in Table 5. Ethanol (99.5%, Chemsol), sodium dodecyl-benzene sulfonate (NaDDBS, Sigma-Aldrich), D.E.R.™ 331 epoxy resin (Dow), RD 108 (Epotec, Thailand) as a reactive diluent for high viscosity epoxy resins, and triethylenetetramine (TETA, Dow) were used as purchased.

**Table 5:** **Properties of the as-received MWCNTs according to the suppliers and literature**

	**From the suppliers**					**Estimated by TEM/SEM (according to ref.** **[** 28 **]** **)**		
**Sample**	**Diameter (nm)**	**Length (μm)**	**Carbon purity (%)**	**Bulk density (kg/m** ^**3**^ **)**	**Surface area (m** ^**2**^ **/g)**	**Average diameter (nm)**	**Average length (μm)**	**Average aspect ratio**
Nanocyl NC7000	9.5	1.5	>90%	66 [63]	250–300	10.0	1.34	134
Baytubes C150P	5-20 (average 11 nm [65])	1-10	>95%	140-160	Not specified	10.5	0.77	73
MWCNT-VAST	10-50 (average diameter 25 nm)	1-10	>90%	Not specified	Not specified	40.1	1.93	48

The average diameter and length of MWCNT-VAST were estimated from the SEM image of the as-received MWCNT powder.

The polymer matrix used was an epoxy resin based on Bisphenol A epichlorohydrin cured by TETA, with a vitrification temperature of around 120°C [72].

### Preparation of MWCNT/epoxy composites via the solution dispersion method

MWCNTs were dispersed in ethanol and the mixture was sonicated at 55°C for 60 min. Then, the epoxy resin (containing 20 wt% of RD 108) was added and the mixture was subjected to continuous simultaneous mechanical stirring and ultrasonication (50 Hz, 300 W) at 55°C for 120 min, followed by solvent evaporation while maintaining mechanical stirring at 80°C. Finally, the hardener (TETA) was added and the matrix was cured under ambient conditions for 24 h before characterization.

### Preparation of MWCNT/epoxy composites via the ball-milling method

MWCNTs were mixed with the epoxy resin (containing 20 wt% of RD 108) and the mixture was subjected to ball-milling using a porcelain vertical style ball mill jar (capacity of 1 L) containing one pivot and 0.5 kg of porcelain balls of 10–20 mm diameters. The milling intensity was 300 rpm, the optimal milling time was 60 min and the weight of each batch was 300 g. After ball-milling, the hardener (TETA) was added and the matrix was cured under ambient conditions for 24 h before characterization.

### Characterization

#### Transmission electron microscopy

The morphology of MWCNT powders and the dispersion of MWCNTs in the cured epoxy matrix was observed by transmission electron microscopy (TEM, JEM 1400, JEOL, Japan) of 70 nm thick microtomed layers of the composites.

#### Raman spectroscopy

Raman spectra were recorded with a Horiba Jobin Yvon HR800 UV spectrometer using an excitation wavelength of 633 nm.

#### Wide-angle powder X-ray diffraction

Wide-angle powder X-ray diffraction (XRD) patterns were recorded at room temperature on a Bruker AXS D8 Advance diffractometer using Cu-Kα radiation (k = 0.15406 nm), at a scanning rate of 0.05 degrees per second. The data were analyzed using DIFRAC plus Evaluation Package (EVA) software. The d-spacing was calculated from peak positions using Cu-Kα radiation and Bragg’s law.

#### Laser diffraction particle size analysis

Laser diffraction particle size analysis was performed on a Horiba LA 920 analyzer, using ethanol as the dispersant. The CNT dispersions in ethanol were prepared at a concentration of 0.5 g/L. Approximately 5–10 mL of the CNT dispersions or 5–10 mg of the CNT powder were introduced into the 100 mL dispersion unit device of the laser particle analyzer for measurements, corresponding to a laser light transmission level between 85-95%. To maintain random orientation of particles in suspension, in-stream 30 watt-ultrasonication (power setting number 3, 1 min) and circulation (level 5) was applied during the measurements.

#### Electrical conductivity measurements

Measurements of electrical conductivities of the samples were performed by a two-probe method using the Keithley Model 2750 multimeter (Keithley Instruments Inc., USA). Samples of 2 × 3 × 0.3 cm were prepared. The pure copper plates which were adhered to the largest surfaces by silver paste (G302-Leitsilber 50 g – Plano GmbH) were then connected to the multimeter to measure the electrical resistance of the samples. The conductivity can be calculated by

$$ \upsigma = 1/\uprho $$

$$ \uprho = \mathrm{R}.\mathrm{A}/\mathrm{L} $$

where ρ is the resistivity (ohm-cm) and R, A and L are the resistance (ohm), cross sectional area (cm^2^) and thickness (cm) of the sample, respectively.

#### Reflection loss measurements

The composite samples for microwave absorption study were fabricated in a single-layered sheet form with dimensions of 150 × 150 × 2–3 mm.

Microwave absorption study at the 8–12 GHz band was performed on a two port vector network analyzer (Anritsu MS2028B; accuracy ± 0.05%, temperature stability ± 1.5 ppm), using a reflection/transmission method. The incident and transmitted waves in the two port vector network analyzer can be mathematically represented by complex scattering parameters (or S-parameters) i.e. S_11_ and S_21_, respectively, which in-turn can be conveniently correlated with reflectance (R) and transmittance (T), i.e. T = |E_T_/E_I_|^2^ = |S_21_|^2^, R = |E_R_/E_I_|^2^ = |S_11_|^2^, giving absorbance (A) as: A = (1-R-T), where E_I_, E_R_ and E_T_ are the power of incident, reflected and transmitted electromagnetic waves respectively. Practically, the reflection was measured at an incident angle of 90°. The electromagnetic wave was incident on the sample backed by metal plate resulting in T ≈ 0. Thus, the reflection loss can be measured as: *RL* = 10log_10_ (1- R).

The measurement uncertainties of the S-parameters and thickness (standard deviations calculated from measurements made on three nominally identical samples) in the frequency range of 8–12 GHz were about 4-5%.

## References

[1] Brosseau C, Quéffélec P, Talbot P (2001). Microwave characterization of filled polymers. J Appl Phys.

[2] Brosseau C (2002). Generalized effective medium theory and dielectric relaxation in particle-filled polymeric resins. J Appl Phys.

[3] Mdarhri A, Carmona F, Brosseau C, Delhaes P (2008). Direct current electrical and microwave properties of polymer-multiwalled carbon nanotubes composites. J Appl Phys.

[4] Mdarhri A, Brosseau C, Carmona F (2007). Microwave dielectric properties of carbon black filled polymers under uniaxial tension. J. Appl. Phys.

[5] Brosseau C, Talbot P (2005). Meas Sci Technol.

[6] Brosseau C, NDong W, Mdarhri A (2008). Influence of uniaxial tension on the microwave absorption properties of filled polymers. J Appl Phys.

[7] Saini P, Choudhary V, Singh BP, Mathur RB, Dhawan SK (2011). Enhanced microwave absorption behavior of polyaniline-CNT/polystyrene blend in 12.4–18.0 GHz range. Synth Met.

[8] Rahmat M, Hubert P (2011). Carbon nanotube–polymer interactions in nanocomposites: a review. Compos Sci Technol.

[9] Breuer O, Sundararaj U (2004). Big returns from small fibers: a review of polymer/carbon nanotube composites. Polym Compos.

[10] Chen H, Jacobs O, Wu W, Rüdiger G, Schädel B (2007). Effect of dispersion method on tribological properties of carbon nanotube reinforced epoxy resin composites. Polym Test.

[11] Garg P, Singh B, Kumar G, Gupta T, Pandey I, Seth RK (2011). Effect of dispersion conditions on the mechanical properties of multi-walled carbon nanotubes based epoxy resin composites. J Polym Res.

[12] Geng Y, Liu MY, Li J, Shi XM, Kim JK (2008). Effects of surfactant treatment on mechanical and electrical properties of CNT/epoxy nanocomposites. Compos Part A: Appl Sci Manuf.

[13] Ma P-C, Mo S-Y, Tang B-Z, Kim J-K (2010). Dispersion, interfacial interaction and re-agglomeration of functionalized carbon nanotubes in epoxy composites. Carbon.

[14] Schmidt RH, Kinloch IA, Burgess AN, Windle AH (2007). The effect of aggregation on the electrical conductivity of spin-coated polymer/carbon nanotube composite films. Langmuir.

[15] Zeng Y, Liu P, Du J, Zhao L, Ajayan PM, Cheng H-M (2010). Increasing the electrical conductivity of carbon nanotube/polymer composites by using weak nanotube–polymer interactions. Carbon.

[16] Müller MT, Krause B, Kretzschmar B, Pötschke P (2011). Influence of feeding conditions in twin-screw extrusion of PP/MWCNT composites on electrical and mechanical properties. Compos Sci Technol.

[17] Bose S, Khare RA, Moldenaers P (2010). Assessing the strengths and weaknesses of various types of pre-treatments of carbon nanotubes on the properties of polymer/carbon nanotubes composites: a critical review. Polymer.

[18] Zhong J, Isayev AI, Huang K (2014). Influence of ultrasonic treatment in PP/CNT composites using masterbatch dilution method. Polymer.

[19] Sandler J, Shaffer MSP, Prasse T, Bauhofer W, Schulte K, Windle AH (1999). Development of a dispersion process for carbon nanotubes in an epoxy matrix and the resulting electrical properties. Polymer.

[20] Song YS, Youn JR (2005). Influence of dispersion states of carbon nanotubes on physical properties of epoxy nanocomposites. Carbon.

[21] Brosseau C, Mdarhri A, Vidal A (2008). Mechanical fatigue and dielectric relaxation of carbon black/polymer composites. J Appl Phys.

[22] Morcom M, Atkinson K, Simon GP (2010). The effect of carbon nanotube properties on the degree of dispersion and reinforcement of high density polyethylene. Polymer.

[23] Gojny FH, Wichmann MHG, Fiedler B, Kinloch IA, Bauhofer W, Windle AH (2006). Evaluation and identification of electrical and thermal conduction mechanisms in carbon nanotube/epoxy composites. Polymer.

[24] Brosseau C, Beroual A, Boudida A (2000). How do shape anisotropy and spatial orientation of the constituents affect the permittivity of dielectric heterostructures?. J Appl Phys.

[25] Aguilar JO RB-QJ, Avilés F (2010). Influence of carbon nanotube clustering on the electrical conductivity of polymer composite films. Express Polym Lett.

[26] Pötschke P, Krause B, Buschhorn ST, Köpke U, Müller MT, Villmow T (2013). Improvement of carbon nanotube dispersion in thermoplastic composites using a three roll mill at elevated temperatures. Compos Sci Technol.

[27] Viets C, Kaysser S, Schulte K. Damage mapping of GFRP via electrical resistance measurements using nanocomposite epoxy matrix systems. Compos Part B: Eng*.* 2014;Doi. 10.1016/j.compositesb.2013.1009.1049.

[28] Castillo FY, Socher R, Krause B, Headrick R, Grady BP, Prada-Silvy R (2011). Electrical, mechanical, and glass transition behavior of polycarbonate-based nanocomposites with different multi-walled carbon nanotubes. Polymer.

[29] Rahaman M, Thomas SP, Hussein IA, De SK (2013). Dependence of electrical properties of polyethylene nanocomposites on aspect ratio of carbon nanotubes. Polym Compos.

[30] Menzer K, Krause B, Boldt R, Kretzschmar B, Weidisch R, Pötschke P (2011). Percolation behaviour of multiwalled carbon nanotubes of altered length and primary agglomerate morphology in melt mixed isotactic polypropylene-based composites. Compos Sci Technol.

[31] Socher R, Krause B, Boldt R, Hermasch S, Wursche R, Pötschke P (2011). Melt mixed nano composites of PA12 with MWNTs: Influence of MWNT and matrix properties on macrodispersion and electrical properties. Compos Sci Technol.

[32] Socher R, Krause B, Hermasch S, Wursche R, Pötschke P (2011). Electrical and thermal properties of polyamide 12 composites with hybrid fillers systems of multiwalled carbon nanotubes and carbon black. Compos Sci Technol.

[33] Qin F, Brosseau C (2012). A review and analysis of microwave absorption in polymer composites filled with carbonaceous particles. J Appl Phys.

[34] Michielssen E, Sajer JM, Ranjithan S, Mittra R (1993). Design of lightweight, broad-band microwave absorbers using genetic algorithms. IEEE Trans Microw Theory Tech.

[35] Fan Z, Luo G, Zhang Z, Zhou L, Wei F (2006). Electromagnetic and microwave absorbing properties of multi-walled carbon nanotubes/polymer composites. Mater Sci Eng B.

[36] Micheli D, Pastore R, Apollo C, Marchetti M, Gradoni G, Primiani VM (2011). Broadband electromagnetic absorbers using carbon nanostructure-based composites. Microwave Theory and Techniques, IEEE Transactions on.

[37] Micheli D, Apollo C, Pastore R, Marchetti M (2010). X-Band microwave characterization of carbon-based nanocomposite material, absorption capability comparison and RAS design simulation. Compos Sci Technol.

[38] Micheli D, Apollo C, Pastore R, Barbera D, Morles RB, Marchetti M (2012). Optimization of multilayer shields made of composite nanostructured materials. Electromagnetic Compatibility, IEEE Transactions on.

[39] Adohi BJ-P, Mdarhri A, Prunier C, Haidar B, Brosseau C (2010). A comparison between physical properties of carbon black-polymer and carbon nanotubes-polymer composites. J Appl Phys.

[40] Balakrishnan A, Saha MC (2011). Tensile fracture and thermal conductivity characterization of toughened epoxy/CNT nanocomposites. Mater Sci Eng A.

[41] Che RC, Peng LM, Duan XF, Chen Q, Liang XL (2004). Microwave absorption enhancement and complex permittivity and permeability of Fe encapsulated within carbon nanotubes. Adv Mater.

[42] Lin H, Zhu H, Guo H, Yu L (2007). Investigation of the microwave-absorbing properties of Fe-filled carbon nanotubes. Mater Lett.

[43] Su Q, Zhong G, Li J, Du G, Xu B (2012). Fabrication of Fe/Fe3C-functionalized carbon nanotubes and their electromagnetic and microwave absorbing properties. Appl Phys A.

[44] Lin H, Zhu H, Guo H, Yu L (2008). Microwave-absorbing properties of Co-filled carbon nanotubes. Mater Res Bull.

[45] Zhang L, Zhu H, Song Y, Zhang Y, Huang Y (2008). The electromagnetic characteristics and absorbing properties of multi-walled carbon nanotubes filled with Er2O3 nanoparticles as microwave absorbers. Mater Sci Eng B.

[46] Zhang L, Zhu H (2009). Dielectric, magnetic, and microwave absorbing properties of multi-walled carbon nanotubes filled with Sm2O3 nanoparticles. Mater Lett.

[47] Deng L, Han M (2007). Microwave absorbing performances of multiwalled carbon nanotube composites with negative permeability. Appl Phys Lett.

[48] Bhattacharya P, Sahoo S, Das CK (2013). Microwave absorption behaviour of MWCNT based nanocomposites in X-band region. Express Polym Lett.

[49] Feng X, Liao G, Du J, Dong L, Jin K, Jian X (2008). Electrical conductivity and microwave absorbing properties of nickel-coated multiwalled carbon nanotubes/poly(phthalazinone ether sulfone ketone)s composites. Polym Eng Sci.

[50] Liu Z, Bai G, Huang Y, Li F, Ma Y, Guo T (2007). Microwave absorption of single-walled carbon nanotubes/soluble cross-linked polyurethane composites. J Phys Chem C.

[51] Qi X, Yang Y, Zhong W, Deng Y, Au C, Du Y (2009). Large-scale synthesis, characterization and microwave absorption properties of carbon nanotubes of different helicities. J Solid State Chem.

[52] Tang N, Zhong W, Au C, Yang Y, Han M, Lin K (2008). Synthesis, microwave electromagnetic, and microwave absorption properties of twin carbon nanocoils. J Phys Chem C.

[53] Silva VA, Folgueras LC, Cândido GM, Paula AL, Rezende MC, Costa ML (2013). Nanostructured composites based on carbon nanotubes and epoxy resin for use as radar absorbing materials. Mater Res.

[54] Savi P, Miscuglio M, Giorcelli M, Tagliaferro A (2014). Analysis of microwave absorbing properties of epoxy MWCNT composites. Prog Electromagn Res.

[55] Musso S, Giorcelli M, Pavese M, Bianco S, Rovere M, Tagliaferro A (2008). Improving macroscopic physical and mechanical properties of thick layers of aligned multiwall carbon nanotubes by annealing treatment. Diamond Relat Mater.

[56] Xu G, Feng ZC, Popovic Z, Lin JY, Vittal JJ (2001). Nanotube structure revealed by high-resolution X-ray diffraction. Adv Mater.

[57] Kosynkin DV, Higginbotham AL, Sinitskii A, Lomeda JR, Dimiev A, Price BK (2009). Longitudinal unzipping of carbon nanotubes to form graphene nanoribbons. Nature.

[58] Marcano DC, Kosynkin DV, Berlin JM, Sinitskii A, Sun Z, Slesarev A (2010). Improved synthesis of graphene oxide. ACS Nano.

[59] Futaba DN, Yamada T, Kobashi K, Yumura M, Hata K (2011). Macroscopic wall number analysis of single-walled, double-walled, and few-walled carbon nanotubes by X-ray diffraction. J Am Chem Soc.

[60] Dresselhaus MS, Jorio A, Saito R (2010). Characterizing graphene, graphite, and carbon nanotubes by raman spectroscopy. Annu Rev Condens Matter Phys.

[61] Singh DK, Iyer PK, Giri PK (2010). Diameter dependence of interwall separation and strain in multiwalled carbon nanotubes probed by X-ray diffraction and Raman scattering studies. Diamond Relat Mater.

[62] Vaisman L, Wagner HD, Marom G (2006). The role of surfactants in dispersion of carbon nanotubes. Adv Colloid Interface Sci.

[63] Krause B, Mende M, Pötschke P, Petzold G (2010). Dispersability and particle size distribution of CNTs in an aqueous surfactant dispersion as a function of ultrasonic treatment time. Carbon.

[64] Krause B, Boldt R, Pötschke P (2011). A method for determination of length distributions of multiwalled carbon nanotubes before and after melt processing. Carbon.

[65] Tessonnier J-P, Rosenthal D, Hansen TW, Hess C, Schuster ME, Blume R (2009). Analysis of the structure and chemical properties of some commercial carbon nanostructures. Carbon.

[66] Kim JA, Seong DG, Kang TJ, Youn JR (2006). Effects of surface modification on rheological and mechanical properties of CNT/epoxy composites. Carbon.

[67] Abu Al-Rub RK, Ashour AI, Tyson BM (2012). On the aspect ratio effect of multi-walled carbon nanotube reinforcements on the mechanical properties of cementitious nanocomposites. Construct Build Mater.

[68] Li J, Ma PC, Chow WS, To CK, Tang BZ, Kim JK (2007). Correlations between percolation threshold, dispersion state, and aspect ratio of carbon nanotubes. Adv Funct Mater.

[69] Seyhan AT, Gojny FH, Tanoğlu M, Schulte K (2007). Rheological and dynamic-mechanical behavior of carbon nanotube/vinyl ester–polyester suspensions and their nanocomposites. Eur Polym J.

[70] Xie X-L, Mai Y-W, Zhou X-P (2005). Dispersion and alignment of carbon nanotubes in polymer matrix: a review. Mater Sci Eng R Rep.

[71] Li G, Xie T, Yang S, Jin J, Jiang J (2012). Microwave absorption enhancement of porous carbon fibers compared with carbon nanofibers. J Phys Chem C.

[72] Toda A, Arita T, Hikosaka M (2000). Kinetic response of an epoxy thermosetting system observed by TMDSC. J Therm Anal Calorim.

